# Synovial fibroblast‐targeting liposomes encapsulating an NF‐κB‐blocking peptide ameliorates zymosan‐induced synovial inflammation

**DOI:** 10.1111/jcmm.13549

**Published:** 2018-01-30

**Authors:** Changcheng You, Jianing Zu, Xiaoqi Liu, Pengyu Kong, Chengchao Song, Rongzhi Wei, Changlong Zhou, Yufu Wang, Jinglong Yan

**Affiliations:** ^1^ Department of Orthopedics Second Affiliated Hospital Harbin Medical University Harbin China

**Keywords:** liposomal nanoparticle, NEMO‐binding domain peptide, nuclear factor‐kappa B, rheumatoid arthritis, synovial fibroblasts

## Abstract

Synovial fibroblasts (SFs) play a crucial role in the inflammatory process of rheumatoid arthritis (RA). The highly activated NF‐κB signal in SFs is responsible for most of the synovial inflammation associated with this disease. In this study, we have developed an SF‐targeting liposomal system that encapsulates the NF‐κB‐blocking peptide (NBD peptide) HAP‐lipo/NBD. HAP‐lipo/NBDs demonstrated efficient SF‐specific targeting in vitro and in vivo. Our study also showed a significant inhibitory effect of HAP‐lipo/NBD on NF‐κB activation, inflammatory cytokine release and SF migration capability after zymosan stimulation. Furthermore, the systemic administration of HAP‐lipo/NBDs significantly inhibited synovial inflammation and improved the pathological scores of arthritis induced by zymosan. Thus, these results suggest that an SF‐targeting NF‐κB‐blocking strategy is a potential approach for the development of alternative, targeted anti‐RA therapies.

## 1 INTRODUCTION

Rheumatoid arthritis (RA) is a chronic autoimmune disease that is characterized by inflammation in the synovium and the destruction of cartilage and bone.^1^ One of the most striking features of RA inflammation is the hyperplasia of synovial fibroblasts (SFs) in the synovial lining. In healthy joints, SFs secrete synovial fluid and extracellular matrix and provide structure to the joint.^2^ However, SFs transform into principal effector cells in RA due to their ability to degrade the extracellular matrix, to provide chemoattractant cytokines and to activate parenchymal cells and infiltrating immunocytes.^2^, ^3^, ^4^

The behaviour of SFs is regulated by multiple intracellular pathways and involves interferon regulatory factors, activator protein‐1, mitogen‐activated protein kinase and the nuclear factor‐kappa B (NF‐κB).^2^, ^5^ The highly activated NF‐κB signal in RA is responsible for the pathological process of RA.^6^ NF‐κB regulates not only pro‐inflammatory genes such as TNF‐α, IL‐6 and IL‐8 but also the transcription of adhesion molecule‐1 and matrix‐degrading enzymes (MMP‐3, MMP‐9, etc.).^7^, ^8^ Moreover, NF‐κB provides a key survival signal that suppresses apoptosis in SFs.^2^ Therapeutic agents targeting NF‐κB have exhibited various degrees of effectiveness in arthritis. However, few of these compounds are SF‐specific, and some deleterious effects have been reported.^9^ Therefore, the development of compounds that target SFs may complement current therapies and avoid major side effects.^10^ Nanoparticles hold significant promise for resolving this challenge as they can be functionalized to confer specific targeting to their encapsulated therapeutic agents.^11^, ^12^ The peptide HAP‐1 (SFsHQFARATLAS) has demonstrated specificity for SFs.^13^ Therefore, delivering nanoparticles coated with HAP‐1 may target NF‐κB inhibition to inflamed joints and reduce systemic toxicity. Currently, multiple steps of NF‐κB activation can be targeted (IKKs, IκBα or p65/p50 subunit) with various available approaches, that is small molecule peptides or nucleic acids.^14^, ^15^ The NEMO‐binding domain (NBD) peptide is a classic NF‐κB inhibitor that can specifically bind to the NEMO domain and interfere with IκB kinase (IKK) complex formation.^16^ Therefore, we hypothesize that liposomes coated with HAP‐1 and loaded with the NBD peptide (HAP‐lipo/NBD) may be able to target and inhibit NF‐κB in SFs of the inflamed synovium, thereby alleviating arthritis. In this study, we describe an SF‐specific liposome with inhibitory activity against NF‐κB and evaluate the therapeutic potential of this nanoparticle in the treatment of inflammatory arthritis.

## 2 MATERIALS AND METHODS

### 2.1 Cell culture

Synovial fibroblasts were obtained from the synovial tissue of patients undergoing total keen arthroplasty who met the American College of Rheumatology classification criteria for RA.^17^ Informed consent was obtained from the patients, and the experiments in this study were carried out according to the World Medical Association Declaration of Helsinki. Isolated synovial tissues were digested, and single‐cell suspensions were obtained as previously described.^18^ The cells were cultured at 37°C with 5% CO_2_ in DMEM supplemented with 2 mmol/L l‐glutamine, 10% FBS, 100 U/mL penicillin and 100 U/mL streptomycin. All the experiments were conducted using synoviocyte cultures from the fourth to seventh passage.

### 2.2 Preparation and characterization of nanoparticles

Peptides used in this study were synthesized by Changxi Biotechnology Company, Shanghai, China. The sequences of the peptides were as follows: HAP‐1, SFHQFARATLAS; NBD peptide, TALDWSWLQTE; and mutant NBD peptide (Mut), TRLDRSWLQTE. The HAP‐1 and NBD peptides were purified to more than 95% purity using high‐pressure liquid chromatography.^19^ The HAP‐lipo/NBD nanoparticles were prepared according to previously described methods.^20^

HAP‐1 peptides were attached to the distal end of the DSPE‐PEG‐maleimide moiety by the formation of a thioether bond between the maleimide‐derivatized PEG and the terminal cysteine on the peptide ligand. The lipid composition of the reaction was optimized during the preparation of the liposomes. For the conjugation of HAP‐1 to the liposomal surface, the peptide was dissolved in HEPES (0.05 mol/L, pH 6.7) and incubated with maleimide‐tagged liposomes overnight (12 hours) at RT in an atmosphere of nitrogen. The liposome‐peptide conjugate was dialyzed for 12 hours to remove unreacted peptide. The amount of conjugated peptide was determined indirectly by analyzing the amount of unreacted peptide in the dialysate after separation from the liposomes. Peptide content in the concentrated aliquots of dialysate was measured spectrophotometrically at 214 nm and by HPLC methods.

The NBD peptide was encapsulated within the internal space of the liposomes. The encapsulation of prednisolone was determined spectrophotometrically by optical density measurements. The NBD peptide was also analysed by analytical HPLC. The amount of NBD peptide incorporated into the liposomes was adjusted to 0.5 mg/mL. The zeta potential and size of the nanoparticles were measured with a zeta potential analyzer (Malvern, UK). The morphology of the nanoparticles was evaluated with transmission electron microscope (TEM; Hitachi H‐7650, Tokyo, Japan). The Mut peptide was also encapsulated, and the concentration was determined and adjusted to the same amount as the NBD peptide using similar methods to those mentioned above.

### 2.3 Confocal fluorescence microscopy

All samples were analysed under a laser scanning confocal microscope (Zeiss LSM510, Germany). For fluorescence analysis, liposomes were labelled with Cy3, and the NBD peptides were labelled by FITC. To investigate the transduction of HAP‐lipo/NBDs in vitro, SFs and MG63 cells were cultured in the presence of the indicated nanoparticles, control peptides or control nanoparticles (containing the NBD peptide at a concentration at 0.05 mg/mL) for 40 minutes. After washing three times, nuclei were stained with 4′,6‐diamidino‐2‐phenylindole (DAPI). These cells were then washed twice and were assessed by fluorescence analysis.

Fluorescence analysis was also employed to investigate the targeting properties of HAP‐lipo/NBDs in vivo. Tail vein injections of 0.5 mL of HAP‐lipo/NBD were performed in zymosan‐stimulated mice (at 24 hours after zymosan injection into the knee). Control animals received injections of 100 μL of PBS into the tail vein. Mice were killed 4 hours after the tail vein injection. The synovial tissues from the knee were removed and fixed in 4% paraformaldehyde. Samples were cut in 20‐μm‐thick sections on a cryostat and stained with DAPI. The sections were then analysed under the confocal microscope.

### 2.4 Electrophoretic mobility shift assays (EMSA)

The NF‐κB probe (5′‐AGTTGAGGGGACTTTCCCAGGC‐3′) was labelled with [γ‐32P] ATP by T4 polynucleotide kinase (Promega) and was purified. Nuclear extract from SFs (20 μg) was mixed with 2 μL of binding buffer and kept on ice for 10 minutes. Then, the labelled probe (0.0175 pmol) was added to the mixture and incubated at room temperature for 30 minutes. A portion of the mixture (20 μL) was loaded onto a 4% native polyacrylamide gel prepared in 0.5× Tris‐borate‐EDTA and was electrophoresed for 2.5 hours. After being dried, the gel was subjected to autoradiography (Bio‐Rad, USA).

### 2.5 Western blot analysis

The SFs were treated with HAP‐lipo/NBDs (NBD peptide concentration at 0.05 mg/mL) for 2 hours and then stimulated with zymosan (0.1 mg/mL) for 45 minutes. The whole cell lysates were separated on SDS‐PAGE gels and then transferred to a PVDF membrane. The blots were usually incubated at 4°C overnight with the primary antibodies anti‐IκBα (1:500 dilution) and anti‐p‐IκBα (1:200) (Santa Cruz, USA). The blots were then incubated with a secondary labelled anti‐rabbit IgG antibody (1:1000) at room temperature for 2‐5 hours. Finally, the proteins were visualized by radiography after reacting with an enhanced chemiluminescence reagent (GE Healthcare, UK). Each blot shown in the figures represents the findings from at least three similar independent experiments.

### 2.6 NF‐κB DNA‐binding activity

The binding of NF‐κB to DNA was measured in nuclear extracts with the TransAM NF‐κB p65 and p50 Assay Kits (Active Motif, Japan). Nuclear extracts were collected using a Nuclear Extract Kit (Active Motif). Proteins were quantified using the BCA method and subjected to an ELISA‐based NF‐κB p65 or p50 transfactor assay. All the procedures were performed according to the manufacturer's instructions.

### 2.7 Cell proliferation assays

The cytotoxicity of HAP‐lipo/NBDs was assessed using a water‐soluble tetrazolium assay (WST‐1; Roch Diagnostics, Germany). SFs (5 × 10^4^ cells/well) were incubated with various concentrations of HAP‐lipo/NBD for 24 hours. The cells were exposed to WST‐1 for 3 hours, and then, the absorbance values were measured at 450 nm.

### 2.8 RT‐PCR

RNA was isolated by the RNAzol (Invitrogen). cDNA was synthesized from total RNA using the PrimeScript RT reagent Kit (TaKaRa, Dalian, China) according to the manufacturer's instructions. Real‐time PCR was performed with SYBR green (Roche, Mannheim, Germany) on a Step One Real‐Time PCR System (Applied Biosystems). Expression data were normalized to β‐actin mRNA expression.

### 2.9 Zymosan‐induced arthritis

Male Wistar rats (8 weeks old) were obtained from the Animal Breeding Center of Second Affiliated Hospital, Harbin Medical University. Animal care and all experiments were conducted in accordance with the guidelines of the animal committee of Harbin Medical University. Rats received an intra‐articular (i.a.) injection of 1 mg of zymosan (Sigma Chemical Company, USA) dissolved in 50 μL of PBS into both of the knee joints. Five animals from the control group received intra‐articular injections of the same volume of PBS. Mice were treated with HAP‐lipo/NBD or PBS (0.5 mL) injections into the tail vein at 8 hours, 1 day, 3 days and 5 days after the zymosan injection. The concentration of the NBD peptide was 0.5 mg/mL. The transverse diameter of the knee joint at the joint space level was measured with digital callipers (Mitsutoyo, Japan) at days 0, 1, 3, 5, 7 and 14. Then, the ratio of the diameters of the arthritic joints to those of the normal joints (from control mice) was calculated.

### 2.10 Histology

Rat knee synovial tissues harvested from dissected knees were fixed in 4% paraformaldehyde, stained with X‐gal and then fixed in 10% formalin. The formalin‐fixed tissues were embedded in paraffin, sliced into 5‐mm sections, stained with X‐gal and counterstained with eosin.

### 2.11 Enzyme‐linked immunosorbent assay (ELISA)

The expression levels of IL‐6 and IL‐8 were determined in the harvested supernatants using corresponding ELISA kits. The synovium of the knee joint was collected, and the expression levels of IL‐1β, IL‐6, MMP9, MMP2 and IL‐8 in the synovial lysates were measured using commercially available ELISA kits (Boster, Wuhan, China) according to the manufacturer's instructions. All samples were assayed in duplicate.

### 2.12 Statistical analysis

Data are expressed as the means ± SD. Differences between multiple groups were evaluated by ANOVA, followed by Dunnett's post hoc test or Tukey comparisons. For analyzing the swelling of the knee joints, repeated measure ANOVA was used to determine statistical significance. The results were considered statistically significant at *P *<* *.05.

## 3 RESULTS

### 3.1 Characteristics of the HAP‐lipo/NBD

**Figure 1:**
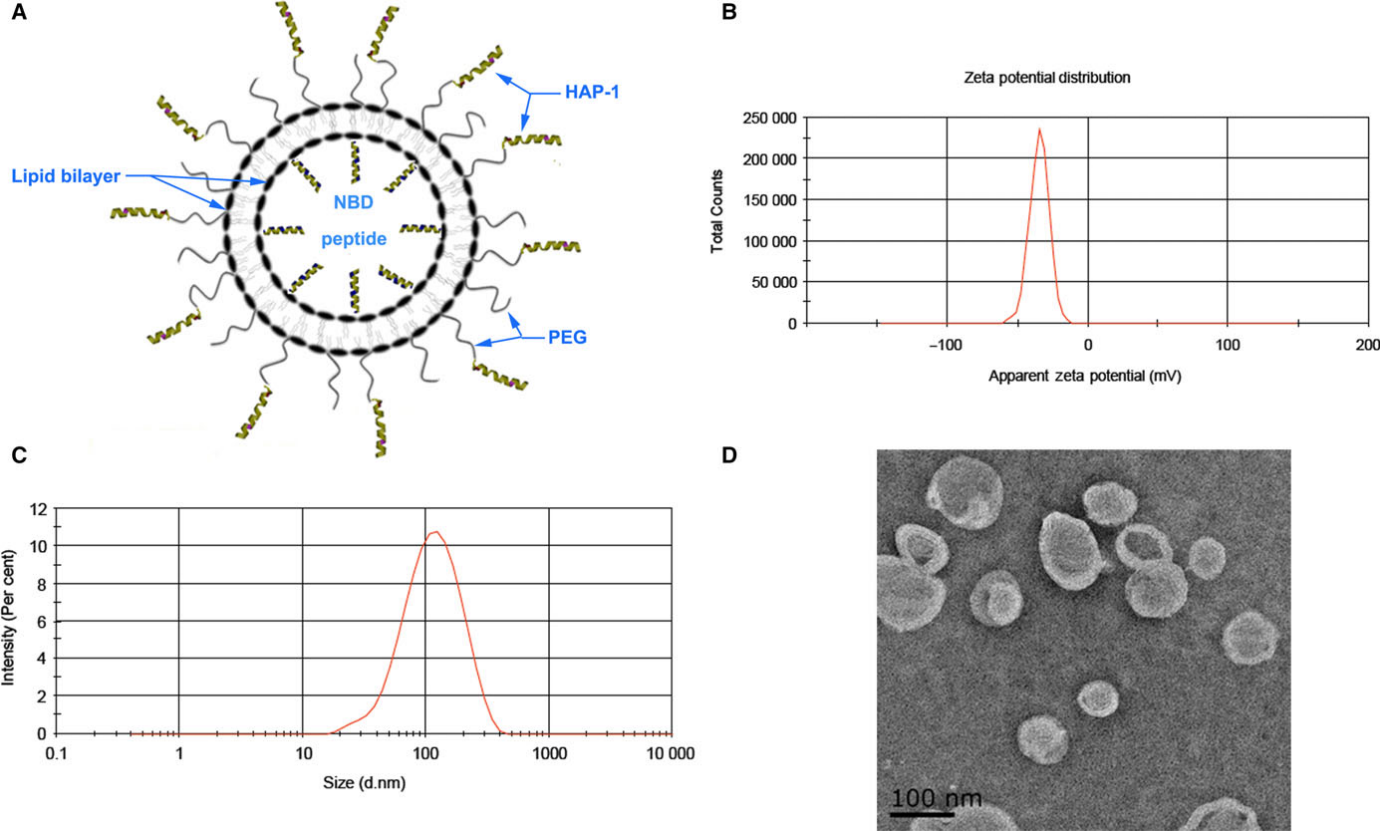
Characteristics of HAP‐lipo/NBD. (A) Schematic diagram of the HAP‐lipo/NBD nanoparticle structure. (B) The zeta potential of the HAP‐lipo/NBD nanoparticles in an aqueous solution was measured by a Malvern Zetasizer Nano instrument. The average zeta potential was −34.6 ± 7.06. (C) The *Z*‐average particle size (d. nm) of the HAP‐lipo/NBD nanoparticles was 97.26 ± 63.06. (D) Transmission electron photomicrographs of the HAP‐lipo/NBD nanoparticles

The targeting polypeptide HAP‐1 (SFHQFARATLAS) was synthesized and covalently conjugated to liposomal nanoparticles encapsulating NBD peptides (Figure 1A). The zeta potential of the HAP‐lipo/NBD was 34.6 ± 7.06 (Figure 1B). Analysis of the HAP‐lipo/NBD nanoparticles by TEM (Figure 1D) revealed that they had a uniform spherical shape with a diameter of 97.26 ± 33.6 nm (Figure 1C).

### 3.2 SF‐specific targeting of HAP‐lipo/NBD in vitro and in vivo

**Figure 2:**
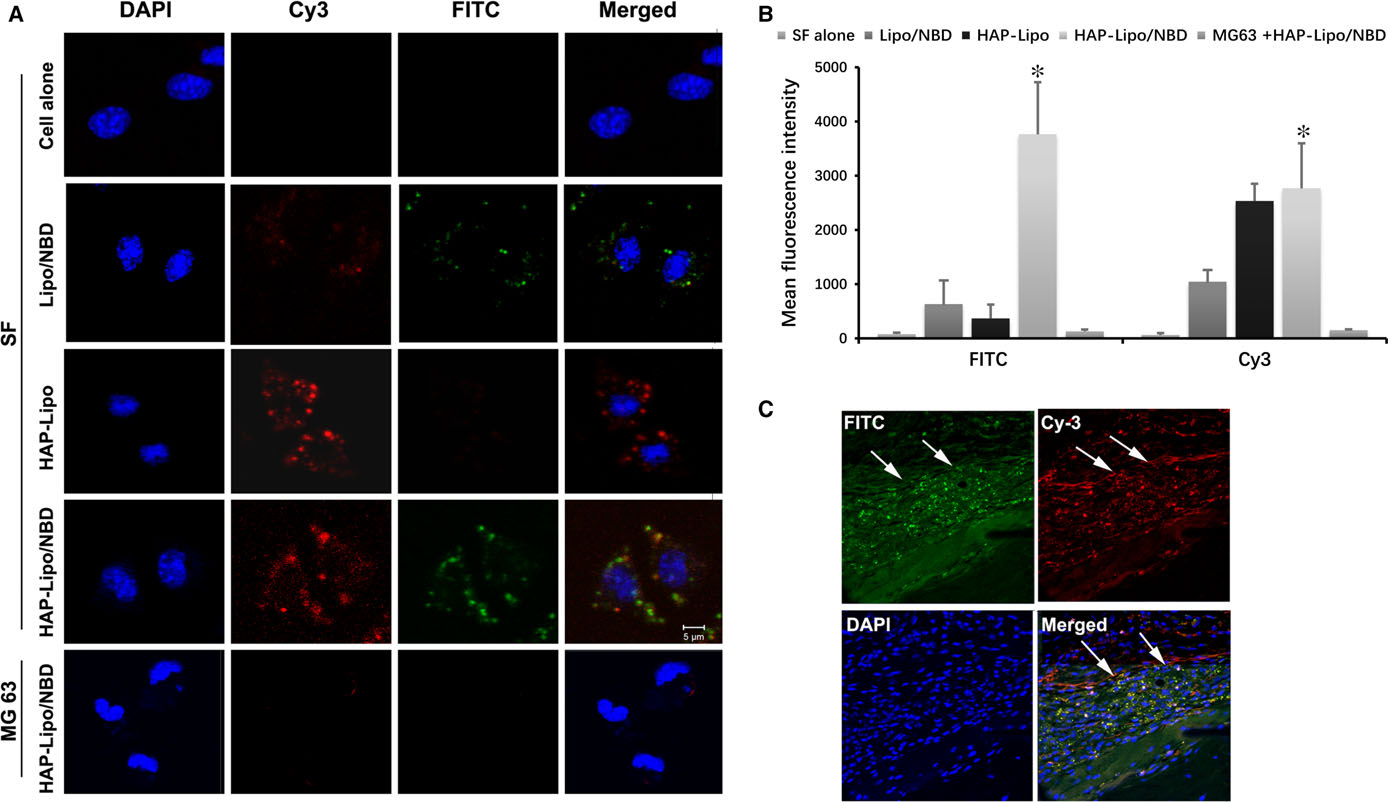
SF‐targeting property of HAP‐lipo/NBD and its accumulation in SFs lining the inflamed synovial membrane. Cell nuclei were stained with DAPI (Blue). Liposomes were labelled by Cy3 (Red), and NBD peptides were labelled by FITC (Green), (A) Representative confocal microscopy images of SFs treated with HAP‐lipo/NBD, HAP‐lipo, Lipo/NBD or PBS control. MG63 cancer cells were also used as a control to assess the SF‐specific targeting property of HAP‐lipo/NBDs. (B) The intensity of the FITC and Cy3 fluorescence was further analysed in vitro. (C) Distribution of HAP‐lipo/NBD in mice. After 4‐h injection intervals of HAP‐lipo/NBD, the synovial membrane of zymosan‐treated mice was removed, and the distribution of HAP‐lipo/NBDs was evaluated by confocal microscopy. The results are expressed as the means ± SD (n = 4). *P <* *.05 HAP‐lipo/NBD‐treated group vs SFs alone group

We proceeded to investigate the in vitro targeting and transmembrane capacity of HAP‐lipo/NBDs. Figure 2A is a confocal image demonstrating the binding of HAP‐lipo/NBDs to SFs, but no binding was observed with the non‐targeted liposomes. HAP‐lipo/NBDs effectively transferred into SFs. To further confirm the SF‐specific targeting of HAP‐lipo/NBDs, similar experiments were conducted in MG‐63 cells. However, HAP‐lipo/NBDs were not transferred into MG‐63 cells. Analysis of the fluorescence intensity of NBDs and liposomes further demonstrated that the transmembrane capability of the liposomal nanoparticles was increased significantly following their modification with HAP (Figure 2B).

In addition, the targeting efficiency of HAP‐lipo/NBD nanoparticles was also tested in vivo. HAP‐lipo/NBDs were administered to zymosan‐induced arthritis model rats via tail vein injection. Analysis of the synovial tissue sections showed that HAP‐lipo/NBDs were mainly localized to the hyperplastic lining layer, which is where SFs usually accumulate in inflammatory conditions (Figure 2C).

### 3.3 HAP‐lipo/NBDs inhibited NF‐κB activation of SFs in vitro

**Figure 3:**
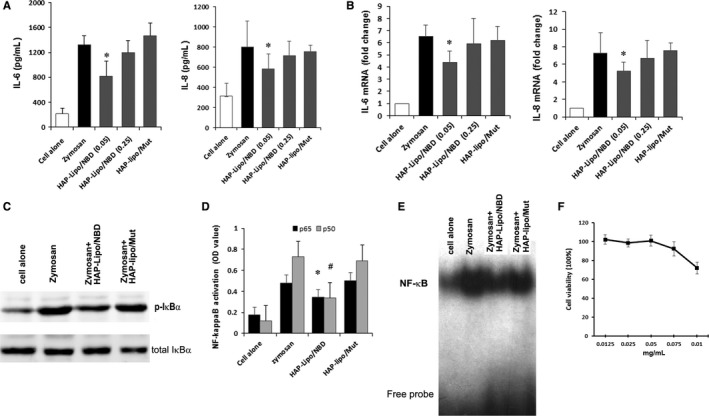
Effect of HAP‐lipo/NBD treatment on the activation of NF‐κB and the production of pro‐inflammatory cytokines. (A) Effects of HAP‐lipo/NBD on the zymosan‐induced production of IL‐6 and IL‐8. SFs were pre‐treated with HAP‐lipo/NBD or HAP‐lipo/Mut and stimulated with zymosan for 24 h. The expression levels of IL‐6 and IL‐8 in culture supernatants were measured by ELISA. (B) Effects of HAP‐lipo/NBD treatment on zymosan‐stimulated mRNA expression levels of IL‐6 and IL‐8. (C) Western blotting results showed that HAP‐lipo/NBD efficiently blocked the zymosan‐induced IKK activity in SFs. (D) The inhibitory action of HAP‐lipo/NBD on the DNA‐binding activity of p65 and p50 was analysed by an ELISA transcriptional kit. (E) Effects of HAP‐lipo/NBD on DNA‐binding activity were measured by EMSA in SFs. (F) Cell proliferation after HAP‐lipo/NBD treatment in SF cultures. Cell proliferation of three SF cultures in the presence of various therapeutic concentrations of NBD peptides was determined by the WST‐1 method. *P <* *.05 HAP‐lipo/NBD‐treated group vs SFs alone group. The results are expressed as the means ± SD (n = 5). ^#^ *P* < .05 zymosan and HAP‐lipo/NBD‐treated group vs zymosan‐treated group

As HAP‐lipo/NBDs specifically targeted SFs and possessed transmembrane capacity, the expression levels of two representative pro‐inflammatory mediators, IL‐6 and IL‐8, in the media from SF cell cultures were measured by ELISA (Figure 3A). Treatment with HAP‐lipo/NBDs (NBD peptide concentration at 0.05 mg/mL) significantly decreased the production of IL‐6 and IL‐8. Furthermore, the mRNA expression levels of IL‐6 and IL‐8 in zymosan‐stimulated SFs were also suppressed after HAP‐lipo/NBD treatment (NBD at 0.05 mg/mL) (Figure 3B). Compared to the control group, no significant changes in IL‐6 or IL‐8 expression were detected after HAP‐lipo/NBD (NBD at 0.025 mg/mL) or HAP‐lipo/Mut treatment.

Next, the effect of HAP‐lipo/NBD (NBD at 0.05 mg/mL) on NF‐κB activity was evaluated in zymosan‐activated SFs. Figure 3C shows that the induction of IκBa phosphorylation was readily detected in cytoplasmic extracts prepared from SFs stimulated with zymosan compared with that of unstimulated SFs. Treatment with HAP‐lipo/NBD (NBD at 0.05 mg/mL) for 6 hours significantly inhibited the phosphorylation of IκBα. Zymosan‐induced NF‐κB p65 and p50 DNA binding were reduced significantly after HAP‐lipo/NBD (NBD at 0.05 mg/mL) treatment for 4 hours (Figure 3D). Furthermore, the EMSA results show that NF‐κB binding was decreased by HAP‐lipo/NBD treatment (NBD at 0.05 mg/mL) in zymosan‐stimulated SFs (Figure 3E). However, treatment with HAP‐lipo/Mut nanoparticles did not affect the zymosan‐induced phosphorylation of IκBα or NF‐κB binding. In addition, HAP‐lipo/NBDs at therapeutic concentrations did not show significant cytotoxicity (Figure 3F). Zymosan stimulation also increased SF adhesion and migration; HAP‐Lipo/NBD treatment inhibited the adhesion and migration capability of zymosan‐stimulated SFs (Supporting information).

### 3.4 HAP‐lipo/NBD inhibited local synovial inflammation in zymosan‐induced arthritis

**Figure 4:**
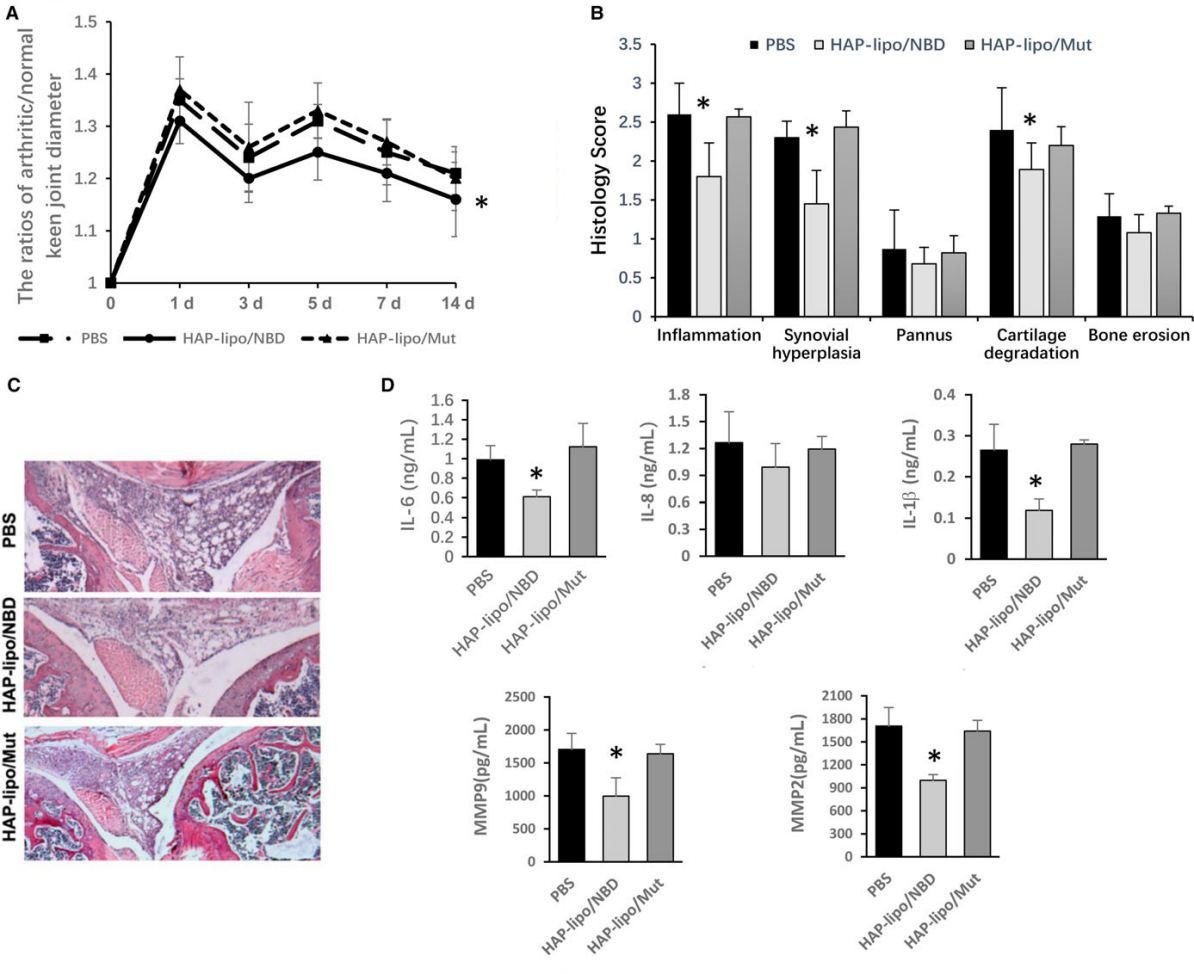
Effects of HAP‐lipo/NBD on zymosan‐induced synovial inflammation in vivo. (A) The effect of HAP‐lipo/NBD on the swelling stimulated by zymosan. PBS, HAP‐lipo/NBD or HAP‐lipo/Mut was injected (500 μL) into the tail vein of mice at different time‐points (8 h, 1 d, 3 d and 5 d) after the zymosan injection. (B) Quantitative microscopic analysis of individual disease metrics from H&E‐stained joint sections from PBS‐, HAP‐lipo/NBD‐ or HAP‐lipo/Mut‐treated mice. (C) Representative images of knees from PBS‐, HAP‐lipo/NBD‐ or HAP‐lipo/Mut‐treated mice at day 7. Representative HE‐stained sections of synovial tissue. The profound accumulation of SFs in the synovial lining was significantly inhibited by the HAP‐lipo/NBD treatment. (D) Effects of HAP‐lipo/NBD on the expression of inflammatory mediators and matrix metalloproteinase. Protein extracts from each experimental group were collected 7 days after the zymosan injection; cytokines were analysed by the ELISA method. The MMP‐9 expression and MMP‐2 expression in joint tissue extracts were analysed by ELISA. The results are expressed as the means ± SD (n = 5). ^#^ *P* < .05 HAP‐lipo/NBD‐treated group vs PBS‐treated group

Based on the above anti‐inflammatory activities of HAP‐lipo/NBD in vitro, we decided to analyse its efficacy in zymosan‐induced arthritis. HAP‐lipo/NBD was administered via tail vein injection to rats with zymosan‐induced arthritis. Mice were treated with tail vein injections of HAP‐lipo/NBD or HAP‐lipo/Mut at 8 hours, 1 day, 3 days and 5 days after the zymosan stimulation. HAP‐lipo/NBD injections significantly inhibited swelling at the different time‐points (Figure 4A). Treatment with HAP‐lipo/NBDs decreased the clinical score of zymosan‐induced arthritis compared with that of animals treated with PBS or the HAP‐lipo/Mut nanoparticles (Figure 4B). Zymosan induced significant cell infiltration, thickening of the synovial membrane, cartilage degradation and bone erosion, all of which were suppressed by HAP‐lipo/NBDs (Figure 4C). Importantly, SF hyperplasia in the lining layer was significantly suppressed by HAP‐lipo/NBD treatment, suggesting that activated SFs are the target of HAP‐lipo/NBDs (Figure 4C). HAP‐lipo/NBD injections significantly inhibited the zymosan‐induced expression levels of inflammatory mediators, including IL‐6, IL‐8 and IL‐1β, and the activity of MMP‐9 and MMP‐2 (Figure 4D), suggesting that the therapeutic effect of HAP‐lipo/NBDs is mediated by targeting NF‐κB activation in inflammation‐stimulated SFs. During our in vivo experiments, HAP‐lipo/NBD treatment did not cause any observable pathological damage to the kidney, liver or heart (data not shown).

## 4 DISCUSSION

Synovial fibroblasts in the synovial lining play a crucial role in producing both cytokines that perpetuate inflammation and proteases that contribute to cartilage destruction.^21^, ^22^ Moreover, increased NF‐κB activity has been linked to the pathological behaviour of SFs.^2^ Therefore, NF‐κB in SFs is a potential target for the development of agents that treat synovial inflammation.^15^ In this study, we demonstrated that the inhibitory effect of HAP‐lipo/NBDs on synovial inflammation is through the targeted suppression of NF‐κB activation in SFs.

Liposomal nanoparticles have emerged as one of the optimal drug carriers for treating RA.^23^, ^24^ PEGylation increases the circulation time and reduces the uptake of liposomes by the liver and spleen, augmenting liposome localization at the inflamed site through the enhanced permeability and retention effect.^11^ A previous study showed that PEG‐coated liposomes, due to their small size (90‐100 nm), are optimal for penetrating inflamed arthritic tissue, resulting in greater therapeutic efficacy.^25^ Importantly, liposomal nanoparticle surfaces can be modified to achieve the selective delivery of encapsulated drug to specific target cells in RA.^11^ Therefore, modified liposomes that target SFs could potentially provide an effective treatment strategy for controlling synovial inflammation.

Increased NF‐κB activity has been linked to various inflammatory disorders.^26^ In RA, NF‐κB is overexpressed in the inflamed synovium. NF‐κB activation is observed in SFs, and both p50 and p65 are highly expressed in the cells of the synovial lining. IKKβ is the primary signalling molecule responsible for the pro‐inflammatory cytokine‐induced IκBα phosphorylation and the subsequent activation of the classical NF‐κB complexes containing the p50 and p65 subunits.^27^ Thus, IKKβ has emerged as an attractive target due to its ability to regulate inflammatory responses in SFs.^2^ The NBD peptide has been shown to block the association of NEMO with the IKK complex, which can inhibit both NF‐κB DNA‐binding activity and target gene expression.^28^ Therefore, the activity of SFs may be efficiently inhibited by the NBD peptide. Our in vitro study demonstrated that HAP‐lipo/NBDs effectively blocked IκBα activation. Blocking IKK activity and IκBα phosphorylation with HAP‐lipo/NBDs also reduced the transcriptional activation of NF‐κB. The effective inhibition of NF‐κB‐mediated DNA‐binding activity was further proven in our experiment. Moreover, the expression levels of genes downstream of NF‐κB, such as IL‐6 and IL‐8, were also inhibited by HAP‐lipo/NBD treatment. These data suggest that it is possible to use this therapy for treating synovial inflammation in vivo.

Several NF‐κB inhibitors have been used to treat inflammatory arthritis. However, non‐specific NF‐κB inhibition may lead to side effects that decrease the drug's therapeutic efficacy.^29^ Glucocorticoids, the most common anti‐RA drugs, are considered to be the most powerful non‐specific inhibitors of NF‐κB but lead to osteoporosis and dysfunction of the hypothalamic‐pituitary‐adrenal axis.^30^ Therefore, there is a clear need for anti‐inflammatory agents that are cell specific and have a lower potential toxicity. To achieve these goals, the “active targeting” technique can be employed. The inflamed synovium is characterized by the accumulation of lymphocytes and inflammatory cells, the activation of SFs and the formation of pannus.^31^ Thus, by coupling targeting ligands to the liposomal membrane, specific cell populations can be targeted at the pathological site. HAP‐1 has been identified as a synovium‐targeting transduction peptide that is capable of facilitating the specific internalization of protein complexes into SFs.^13^, ^32^ Therefore, covalently linking HAP‐1 to the surface of PEGylated liposomes can result in the binding of these targeted liposomes to SFs. Thus, therapeutic liposomes targeting SFs can be achieved with HAP‐modified liposomes that are loaded with therapeutic agents. Previous studies have shown that immunosuppressive, peptide‐loaded liposomes are effective in ameliorating arthritis.^20^ In this study, using systematic treatments, we demonstrated the SF‐specific targeting property of HAP‐lipo/NBDs in vitro and the accumulation of HAP‐lipo/NBDs in the lining of the synovium. Additionally, systemic injections of HAP‐lipo/NBDs did not lead to significant toxicity, which is possibly due to its cell‐specific targeting and lower dose of therapeutic peptide. These data suggest that HAP‐lipo/NBD is an SF‐targeting liposome that incorporates NBD peptides, a potential therapeutic agent for ameliorating synovial inflammation.

Zymosan‐induced arthritis exhibits the same characteristics as RA, with pro‐inflammatory cytokines, inflammatory cell infiltration and hyperplasia being extensively involved in the pathogenesis of this disease.^33^, ^34^ In the inflamed joint, SFs are major cytokine producers. The complex inflammatory network induces diverse effects including the stimulation of acute phase responses and the differentiation of osteoclasts and lymphocytes.^35^ NF‐κB plays a key role in the regulation of inflammatory genes in SFs.^7^ In this study, HAP‐lipo/NBDs suppressed the zymosan‐induced synovial inflammation, as demonstrated by reduced levels of NF‐κB targets, including IL‐6, IL‐8 and IL‐1β. The resulting decrease in inflammatory cytokines further reduced the lymphocyte and neutrophil infiltration into the synovium and synovial cavity. MMP‐9 is an important mediator that leads to RA synovial fibroblast survival, proliferation, migration and invasion.^36^ Thus, the inhibited production of MMP‐9 in the in vivo study suggests that HAP‐lipo/NBD is an effective agent for disrupting the SF‐mediated bone‐harming behaviour.^37^ Furthermore, the pathological analysis showed that HAP‐lipo/NBDs effectively inhibit cell hyperplasia in the synovial lining. The suppressed SF hyperplasia may be attributed to the disruption of inflammatory networks.

In summary, our study shows that HAP‐lipo/NBDs, SF‐targeting liposomes that incorporate an NF‐κB blocking peptide, inhibit NF‐κB activity in SFs and efficiently attenuate the zymosan‐induced synovium inflammation. These results suggest that the SF‐targeting, NF‐κB blocking strategy is a potential approach for the development of targeted inhibitors as alternative anti‐arthritic therapies.

## CONFLICT OF INTERESTS

The authors confirm that there is no conflict of interests.
