# Supplementary material for: Synovial fibroblast‐targeting liposomes encapsulating an NF‐κB‐blocking peptide ameliorates zymosan‐induced synovial inflammation
Source: J Cell Mol Med. 2018 Jan 30;22(4):2449–57. doi: 10.1111/jcmm.13549 (PMC5867099; doi:10.1111/jcmm.13549)
Supplement: Supplementary file 1 [file JCMM-22-2449-s001.docx]

**Supplementary Methods：**

**Adhesion assay and Cell migration assay.**

This assay was performed on plates coated with human fibronectin (10 μg/ml in PBS) for 2 h at 37°C. In brief, cells were allowed to adhere to the substrate for 4 h at 37°C, and unbound cells were removed by washing twice with PBS. Adhered cells were then stained with 0.2% crystal violet in 10% ethanol for 10 min at room temperature. After washing with water, cells were lysed with 33% acetic acid, and their absorbance was determined at 570 nm using a SpectraMax Plus photometer (Molecular Devices).

Cell migration assay. Modified Boyden chambers with 8-μm pores (Corning) were coated at the lower surface with 10 μg/ml human fibronectin for 2 h at 37°C. Cells were harvested with trypsin/EDTA from nonconfluent cultures, washed with PBS, and resuspended to 106 cells per ml. The cell suspension was then added to the upper chamber, and the cells were allowed to migrate at 37°C, 5% CO2, for 4 h. Nonmigratory cells were removed from the upper surface of the membrane, whereas migratory cells in the lower compartment of the chambers were washed with PBS and stained with 0.2% crystal violet in 10% ethanol for 10 min. After extensive washing in H2O, the cells were lysed, and absorbance was measured at 570 nm with a SpectraMax Plus photometer.

**Results: The effect of HAP-lipo/NBD on adhesion and migration activity of SFs.**
